# Examining associations between medical fear and perceptions of provider trust, provider empathy, and medical mistrust among college-attending young adults

**DOI:** 10.1371/journal.pone.0321412

**Published:** 2025-06-25

**Authors:** Sarah C. M. Morton, Ashley Miller, Robin S. Everhart

**Affiliations:** Department of Psychology, Virginia Commonwealth University, Richmond, Virginia, United States of America; Kasturba Medical College, Manipal Academy of Higher Education (MAHE), INDIA

## Abstract

**Objective:**

The current study examined associations between various dimensions of medical fear (e.g., blood, mutilation, sharp objects, injection/blood draws, examinations/symptoms) and perceptions of provider trust, provider empathy, healthcare system mistrust, and attitudes toward medical care-seeking. Additionally, we explored the associations between different dimensions of medical fear and medical care engagement.

**Methods:**

A convenience sample of 479 young adults (18–26 years) attending a large, urban Mid-Atlantic university completed a cross-sectional online survey during the fall of 2022 assessing medical fears, provider trust, perceptions of provider empathy, medical care-seeking attitudes, and medical mistrust. Participants with medical fears (*n* = 211) answered an additional open-ended question regarding medical care engagement. Multiple regression models were used to examine associations between medical fear dimensions and outcome measures. A binary logistic regression was performed to examine the likelihood of health care engagement based on different medical fear dimensions. Statistical significance was set at p < .05.

**Results:**

Participants identified as 75.8% female (*n* = 363), 47.0% White (*n* = 219), and 25.7% (*n* = 122) reported having a chronic illness. Increasing levels of mutilation fear were significantly associated with lower ratings of provider trust (β = −.286, *p* < .001) and empathy (β = −.172, *p* = .010), as well as more medical mistrust (β = .227, *p* < .001). None of the five medical fear dimensions were significantly associated with medical engagement.

**Conclusions:**

Findings highlight the role of mutilation fears in patient-provider relationships and views about the healthcare system in general. While these fears were not associated with medical care avoidance in our study, their association with patient-provider relationships may have implications for adherence to medical recommendations and health outcomes. Patient-centered collaborative care that takes medical fears into consideration may help strengthen patient-provider relationships and mitigate potential negative health outcomes.

## Introduction

Medical fears are common (e.g., needle fear affects approximately 63% of adults worldwide [1]) and can involve the experience of fear, disgust, and anxiety related to various medical stimuli (e.g., injury/illness, needles/injections, blood, mutilation). For roughly 4% of the U.S. adult population [2], and an even higher percentage of young adults [3], these fears are so severe that they manifest as a specific phobia called blood-injection-injury phobia (BII phobia). Specific phobias involve persistent fear/anxiety of certain stimuli and avoidance or tolerance of the stimuli with significant distress [4]. Unlike other specific phobias, BII phobia and medical fears can have significant implications for engagement in medical care. Specifically, affected individuals report blood donation refusal, vaccine hesitancy, and delay/avoidance of preventive health procedures and even necessary medical care [1,5,6].

Although fear, disgust, and anxiety are inherently characterized by avoidance, perceptions of health care provider empathy and trust have the potential to positively impact health outcomes and medical recommendation compliance [7,8]. In the context of medical fears, a case study of a pregnant woman with needle phobia found that trusting patient-provider relationships helped foster a greater sense of control for the patient and decreased their experience of fear during medical procedures throughout pregnancy [9]. Similarly, a recent study in a sample of young children with type 1 diabetes found that trusting patient-provider relationships were associated with fewer symptoms of needle phobia [10]. In addition to provider trust, perceptions of provider empathy have also been found to reduce BII phobia symptoms. Specifically, there is evidence to suggest that patients with a history of venipuncture-related syncope (i.e., fainting during blood draws) are less likely to faint when providers employ empathic interventions (e.g., normalizing fear, praising patient bravery) compared to normal blood draw procedures [11]. Conversely, factors like healthcare system mistrust are associated with lower intention to seek medical care [12].

Although healthcare systems and providers can play an important role in helping those with medical fears better engage in appropriate procedures/treatment, research examining current clinical practices suggests that providers frequently underestimate medical fears (e.g., needle anxiety) and that patients often feel that their fears are inadequately addressed [1,13]. In fact, shame and stigma associated with medical fears like needle phobia is common in adult populations, with some patients reporting that their fear is trivialized, mocked, or dismissed by medical providers [14]. Lack of provider awareness and intervention (e.g., empathy, use of pain management techniques) may decrease patients’ perceptions of provider empathy – a factor which significantly impacts the patient-provider relationship and patient trust in their provider [15]. In turn, this may contribute to poor health outcomes due to decreased engagement in preventive healthcare and decreased trust in the healthcare system overall.

### Gaps in the literature

There is some evidence that provider trust and empathy may be associated with reduced stress and better medical care engagement in populations impacted by medical fears [9,10]. However, studies examining these associations are uncommon and focus solely on one dimension of medical fears (i.e., injections/needles). Similarly, most research examining fear-based medical avoidance exclusively feature needle fear (e.g., vaccine uptake, blood draws) [1,16]. Although the COVID-19 pandemic has led to a significant uptick in research on the role of needle fear in adult vaccine hesitancy and needle fear treatments [16–18], the focus does not comprehensively examine medical fears or BII phobia. As such, it remains unclear how different aspects of medical fears may relate to medical care engagement and perceptions of provider trust, empathy, and medical mistrust.

Despite evidence of insufficient responses by healthcare providers, medical fear literature often attributes medical avoidance to patients’ experiences of fear or disgust. Moreover, other pathways potentially impacting medical engagement (e.g., perceptions of provider empathy, provider/healthcare system mistrust) remain underexplored. Therefore, the current study examined associations between various dimensions of medical fear and participant reports of provider trust, provider empathy, healthcare system mistrust, and attitudes toward medical care-seeking. Additionally, we explored the associations between different dimensions of medical fear and medical care engagement. It was hypothesized that higher scores across the five dimensions of medical fear would be associated with lower ratings of provider trust and empathy, greater mistrust of the healthcare system, and unfavorable attitudes toward medical care-seeking.

### Data transparency and openness

We report information on our sample size determinations, data manipulations, and measures used in this study. Our data and study materials are publicly available at Open Science Framework (DOI https://doi.org/10.17605/OSF.IO/7GMXD): https://osf.io/7gmxd/.

## Materials and methods

### Design

This was a cross-sectional study design utilizing data from an anonymous online survey.

### Participants and sampling method

A convenience sample of U.S. college students (*n* = 479) attending a large, urban university in Richmond, Virginia were recruited between July 25, 2022 and December 25, 2022. The study was advertised to undergraduate students through the university SONA Systems platform, an online research participation system used to advertise university studies to student participant pools and record study participation. The participant pool for this study featured mostly undergraduate students majoring in psychology. Interested participants clicked a link in the SONA study description and were redirected to Qualtrics, an online platform used to distribute surveys and collect participant responses, where they reviewed the study information sheet and completed online questionnaires.

To be included in the study, participants had to be between 18–26 years old, enrolled at least part-time at the university, and comfortable reading and communicating in English. The current study targeted young adults (18–26 years) for recruitment due to the higher prevalence rate of BII phobia when compared to older adults [3]. Children and adults outside of the young adult range (i.e., 27 + years old) were excluded from the current study. Non-college attending young adults were also excluded due to the unique population differences between college and non-college attending young adults. Specifically, college students were selected due to university requirements for vaccinations (including recent COVID-19 vaccines) to enroll in studies or return to campus following the COVID-19 pandemic. This inclusion criterion was selected to hopefully increase the likelihood of more recent needle experiences and facilitate recall when answering study questions about patient-provider interactions.

### Measures

#### Demographics.

Participants reported on their gender, age, race, ethnicity, chronic illness status, family income, employment, and health insurance coverage.

#### Medical fears.

The Medical Fear Survey – short version (MFS-SV) [19,20] is a 25-item questionnaire that presents a series of scenarios about five different dimensions of medically-related fears: blood (e.g., “seeing a small vial of human blood”), injections/blood draws (e.g., “having blood drawn from your arm”), sharp objects (e.g., “handling a butcher knife”), medical examination/symptoms (e.g., “feeling nauseated”), and mutilation (e.g., “observing a surgical amputation”). Respondents rated their fear of each scenario on a scale of 0–3 (0-no fear or concern at all to 3-intense fear) and totals are calculated for each subscale. Subscale totals are summed to produce an overall scale score with higher scores indicating greater fear. Validation studies have demonstrated acceptable internal consistency across all subscales of the MFS-SV (0.74–0.94), as well as strong construct validity and the ability to distinguish between high and low levels of BII phobia symptoms [19]. In this sample, Cronbach’s alpha for the total scale was α = .91 (see Table 2 for subscales).

**Table 1:** Comparative demographics for subsamples with (*n* = 211) and without medical fears (*n* = 267).

Variable			Total Sample n (%)	Medical Fear n (%)	No Medical Fear n (%)	Chi-square
Gender Identity						*X*^2^ (2, *N* = 477) = 6.91*
	Female		363 (75.8%)	164 (77.7%)	199 (74.5%)	
	Male		98 (20.5%)	35 (16.6%)	63 (23.6%)	
	Other		18 (3.7%)	12 (5.7%)	5 (1.9%)	
Race						*X*^2^ (3, *N* = 465) = 5.32
	White/ Anglo American		219 (47.0%)	107 (51.7%)	111 (43.0%)	
	Black/ African American		110 (23.6%)	41 (19.8%)	69 (26.7%)	
	Asian		99 (21.2%)	40 (19.3%)	59 (22.9%)	
	Other		38 (8.2%)	19 (9.1%)	19 (7.4%)	
Ethnicity						*X*^2^ (1, *N* = 473) =.622
	Latinx		81 (17.1%)	39 (18.7%)	42 (15.9%)	
Employment Status						*X*^2^ 2, *N* = 472) =.882
	Full-time		19 (4.0%)	10 (4.7%)	9 (3.4%)	
	Part-time		234 (49.2%)	106 (50.2%)	127 (48.1%)	
	Other		223 (46.8%)	95 (45.0%)	128 (48.5%)	
Health Insurance Status						*X*^2^ (1, *N* = 475) = 7.06†
	Insured		438 (92.0%)	201 (95.7%)	236 (89.1%)	
	Not Insured		38 (8.0%)	9 (4.3%)	29 (10.9%)	
Health Insurance Adequacy						*X*^2^ (1, *N* = 434) =.971
	Coverage meets needs		403 (84.1%)	187 (94.0%)	215 (91.5%)	
	Coverage does not meet needs		32 (6.7%)	12 (6.0%)	20 (8.5%)	
Family’s Annual Household Income						*X*^2^ (5, *N* = 472) = 7.27
	$200,000 and up		59 (12.5%)	27 (13.0%)	32 (12.1%)	
	$100,000 - $199,999		145 (30.7%)	71 (34.1%)	74 (28.0%)	
	$60,000 - $99,999		133 (28.1%)	61 (29.3%)	72 (27.3%)	
	$30,000 - $59,999		66 (14.0%)	26 (12.5%)	39 (14.8%)	
	$15,000 - $29,999		44 (9.3%)	17 (8.2%)	27 (10.2%)	
	Less than $14,999 per year		26 (5.5%)	6 (2.9%)	20 (7.6%)	
Chronic Health Status						*X*^2^ (1, *N* = 473) = 6.78†
	No Chronic Illness		352 (73.5%)	144 (68.6%)	208 (79.1%)	
	Experiences Chronic Illness		122 (25.7%)	66 (31.4%)	55 (20.9%)	

*Note*: For gender, non-binary and transgender responses were coded as “Other.” For race, Mixed/Multi-racial, Native Hawaiian or Other Pacific Islander, and American Indian/Alaskan Native responses were coded as “Other.” For employment status, responses of retired and “other” status were coded as “Other.”

*p < .05, †*p* < .01.

**Table 2:** Descriptive statistics.

	M	SD	Range	Skew	Kurtosis	Cronbach’s α
Medical Fear Survey Total	21.47	12.17	0-67	.664	.277	.911
Injections & Blood Draws	3.26	2.93	0-12	.748	−.247	.829
Examinations & Symptoms	6.27	3.71	0-17	.405	−.131	.808
Sharp Objects	2.79	3.05	0-14	1.17	.872	.834
Blood	2.31	2.85	0-12	1.37	1.23	.847
Mutilation	6.85	3.85	0-15	.049	−848	.816
Provider Empathy	24.71	6.25	5-35	−.259	−.452	.904
Medical Mistrust	29.56	5.68	9-45	−.109	.584	.833
Provider Trust	36.08	7.25	15-50	−.252	−.258	.523
Intention to Seek Medical Care	24.66	6.93	1-36	−.521	−.132	.861

*Note*: M = Mean, SD = Standard deviation.

#### Provider trust.

Participants completed the Wake Forest Physician Trust Scale (WFPTS) [21], which measures one’s trust in their medical provider. Respondents rated ten statements about their provider (e.g., “your doctor is extremely thorough and careful”) using a 5-point Likert scale (1-strongly disagree to 5-strongly agree). Items were summed to generate a total score. Higher scores indicated greater trust in the medical provider. The original validation study revealed good convergent validity, high internal consistency (0.93), and good test-retest reliability (0.75) [21]. In this sample, Cronbach’s alpha was α = .52.

#### Provider empathy.

The Jefferson Scale of Patient Perceptions of Physician Empathy [22] was used to assess perceptions of provider empathy during a recent medical appointment. Participants read five statements (e.g., “understands my emotions, feelings and concerns”) about their perception of providers and responded using a 7-item Likert scale (1-strongly disagree to 7-strongly agree). Higher scores reflected higher perceptions of provider empathy. The initial validation study described good face, content, and convergent validity, as well as high internal consistency (α = 0.91–0.96) [22]. Cronbach’s alpha was α = .90 in this sample.

#### Intention to seek medical care.

Participants completed the Attitudes Toward Seeking Medical Care: Action/intention sub***-***scale [23,24], which is a 12-item measure assessing medical help-seeking attitudes (e.g., “I would rather live with some physical problems than go through a lot of medical tests and check-ups”) using a 4-point Likert scale (0-agree to 3-disagree). Items were summed to generate a total attitudes score. Higher scores indicated greater intention to seek medical care. Validation studies have yielded high internal consistency (0.82–0.90), good congruent validity, as well as good test-retest reliability (0.85) and predictive validity (i.e., correlation between medical attitude and medical contact) of the primary action-intention subscale [23,24]. Cronbach’s alpha for this subscale was α = .86.

#### Medical mistrust.

The Revised Health Care System Distrust Scale was used to measure medical mistrust across the domains of perceived technical competence and provider values. Respondents rated their agreement with nine statements (e.g., “the health care system makes too many mistakes”) using a 5-point Likert scale (1-strongly agree to 5-strongly disagree) [25]. Items were summed to yield a total overall score ranging from 9−45, with higher scores reflecting greater medical mistrust. Results of the validation study yielded high internal consistency for the overall score (Cronbach’s α = 0.82–0.87) [25]. For this sample, Cronbach’s alpha for the total scale was α = .83.

#### Medical engagement.

Participants answered a yes/no question asking if they experienced medical fears. Respondents who answered “yes” answered an additional open-ended question regarding medical care engagement (e.g., “Have you ever had issues going to see a healthcare provider or following medical recommendations because of your fears?”). Open-ended responses were then coded as “yes” or “no.”

### Statistical analyses

Data were analyzed using IBM SPSS statistics software (Version 28). Prior to running analyses, all data were checked for missingness, normality, linearity, homoscedasticity, and multicollinearity following statistical methods outlined by Tabachnick and Fidell [26]. Assumptions for one-way ANOVA and all regression models were met. For ANOVA analyses, normality was assessed by plotting residuals and equal variance was tested by comparing the smallest and largest standard deviations for each factor level to ensure the ratio between these deviations fell within the accepted range of 0.5 to 2. For covariate testing, a one-way ANOVA was used to examine differences in outcome variables by race. *Post hoc* analyses using Tukey’s HSD test further specified differences by race. Independent samples t-tests were used to assess for differences in outcome variables by chronic health status (present vs. not-present), ethnicity (Latinx/Spanish origin), and gender. Correlational analyses were run to assess for associations between age and outcome variables.

An a priori power analysis was conducted using G*Power v. 3.1.9.7 to determine the necessary sample size for the current study for each of the main hypotheses [27]. In the present study, assuming a small-to-medium effect size, 453 participants should be adequate to detect an effect for the main outcome analyses (power = .80, α = .05). To allow for incomplete survey responses and data missingness, an additional 26 participants were recruited.

For hypothesis testing, multiple regression models were used to examine associations between the five MFS-SV dimensions (injections/blood draws, sharps, mutilation, examination/symptoms, and blood) and outcome measures of provider trust, provider empathy, mistrust of the healthcare system, and attitudes toward seeking medical care. A binary logistic regression was performed in the subsample of participants with medical fears to examine the effects of the five MFS-SV dimensions on the likelihood that students would either avoid or engage in medical care. For all regression analyses, predictors and covariates were entered simultaneously into the models. We were adequately powered for all analyses. For all analyses, statistical significance was set at p < .05.

### Ethical considerations

This study received ethical approval from the university Institutional Review Board (IRB; IRB Registration Number IRB00000410) on June 23, 2022 (Approval # HM20024402). This study was approved for an exempt review and no identifying participant information was collected. Participants received information about the study and were asked to select whether or not they wished to participate, but consent documentation was not required.

## Results

### Demographics and descriptives

Of the participants enrolled (*N* = 479), 478 were included in analyses; one was excluded due to inconsistent response patterns. Participants were 18–26 years old (*M* = 18.78, *SD* = 1.26). Participants identified as 75.8% female, 47.0% White, and 25.7% reported having a chronic illness (Table 1). Chi-squared tests were conducted to determine if demographic characteristics varied significantly between those with medical fears (*n* = 211) compared to those without (Table 1). Results indicated that those with medical fears were more likely to be female, experience chronic illness, and have health insurance than those without medical fears. In the medical fears subsample, 33.0% endorsed a history of fear-related medical avoidance. Additional descriptive statistics are presented in Table 2.

### Preliminary analyses

On the sharps and blood subscales of the MFS-SV scale, 12 univariate outliers were winsorized [26]. Two multivariate outliers were retained as their scores were likely elevated due to intense fear. After winsorizing, there was still a slight positive skewness and kurtosis for both the sharps and blood subscales (Table 2). As previously noted, one participant was excluded from analyses due to inconsistent responses to bring the final sample size to 478.

### Covariate testing

One-way ANOVA results indicated that significant differences by race were found on the blood draws/injections subscale, *F*(3, 448) = 3.09, *p* = .027. Tukey’s HSD *post hoc* tests revealed that White students (*M* = 3.59, *SD* = 2.94) endorsed significantly more fear of blood draws/injections than Asian students (*M* = 2.56, *SD* = 2.59; *p* = .018). Correlational analyses indicated that age was not significantly associated with any of the outcome variables or medical fears. Independent samples t-tests suggested that students with chronic illnesses (*M* = 30.71, *SD* = 5.40) endorsed significantly more medical mistrust than those without chronic illnesses (*M* = 29.12, *SD* = 5.69), *t*(457) = 2.63, *p* = .009. While scores on outcome variables did not vary by gender, female students (*M* = 22.59, *SD* = 12.18) endorsed significantly more symptoms of medical fears than males (*M* = 17.02, *SD* = 11.64; *t*(428) = −3.91, *p* < .001). Lastly, results of independent samples t-tests indicated that outcome variables did not significantly vary based on ethnicity. Race and chronic health status were controlled for across all analyses. Although our outcome variables did not vary significantly by ethnicity or gender, they were also included as covariates due to their association with these variables in the literature [28–30].

### Analysis of main hypotheses

Multiple regression models examining the five dimensions of medical fears (IV) and provider trust (DV) revealed that the overall model was significant, *F*(11, 405) = 2.30, *p* = .010, *R*^2^ = .06. Fears related to medical examinations/symptoms, *t*(405) = 1.98, *p* = .048, mutilation, *t*(405) = −4.38, *p* < .001, and sharp objects, *t*(405) = 1.97, *p* = .049, were significan*t* predictors of provider trust. Specifically, it was observed that greater fear of mutilation was associated with lower levels of provider trust (β = −.286). Conversely, greater fear of sharp objects (β = .121) and examinations/symptoms (β = .121) were associated with higher levels of provider trust. Fear of blood and injections/blood draws were not significant predictors of provider trust.

The regression model examining the impact of medical fears on medical mistrust showed that the overall model was significant, *F*(11, 396) = 4.16, *p* < .001, *R*^2^ = .11. Fear of sharp objects, *t*(396) = −2.57, *p* = .010, mu*t*ilation, *t*(396) = 3.53, *p* < .001, and blood, *t*(396) = 2.17, *p* = .031, significantly predic*t*ed medical mistrust, while the remaining two subscales did not. Results further indicated that greater fear of mutilation (β = .227) and blood (β = .144) was associated with higher levels of medical mistrust, while greater fear of sharp objects was associated with lower levels of medical mistrust (β = −.156).

The overall model with provider empathy as an outcome was not significant. However, fear of mutilation significantly predicted perceptions of provider empathy, *t*(403) = −2.60, *p* = .010. Specifically, more fear of mutilation was associated with less provider empathy (β = −.172). The remaining four subscales were not significant predictors. The last multiple regression analysis assessing the impact of medical fears on intention to seek medical care was not significant (see Table 3).

**Table 3:** Multiple regression results of medical fear dimensions on provider trust, provider empathy, and medical mistrust.

Predictor Variable Outcome Variable	*B*	*SE B*	*β*	*t*	*p*
Mutilation					
Provider Trust	−.535	.122	−.286	−4.38†	<.001
Provider Empathy	−.282	.108	−.172	−2.60*	.010
Med. Seeking Attitude	−.188	.121	−.105	−1.55	.121
Medical Mistrust	.334	.095	.227	3.53†	<.001
Blood					
Provider Trust	−.113	.171	−.045	−.659	.510
Provider Empathy	−.141	.151	−.064	−.933	.351
Med. Seeking Attitude	.104	.168	.044	.623	.534
Medical Mistrust	.284	.131	.144	2.17*	.031
Examination/Symptoms					
Provider Trust	.236	.119	.121	1.98*	.048
Provider Empathy	.154	.106	.090	1.45	.147
Med. Seeking Attitude	.100	.117	.054	.855	.393
Medical Mistrust	−.081	.092	−.053	−.881	.379
Injections and Blood Draws					
Provider Trust	.043	.148	.018	.292	.770
Provider Empathy	.021	.131	.010	.158	.874
Med. Seeking Attitude	−.149	.146	−.064	−1.02	.307
Medical Mistrust	.111	.114	.058	.976	.330
Sharp Objects					
Provider Trust	.288	.146	.121	1.98*	.049
Provider Empathy	.240	.129	.116	1.86	.064
Med. Seeking Attitude	−.106	.143	−.047	−.740	.459
Medical Mistrust	−.289	.112	−.156	−2.57*	.010

*Note:* All analyses controlled for gender, race, ethnicity, and chronic health status. B = unstandardized beta coefficient, SE B = standard error of unstandardized beta, β = standardized beta coefficient, t = t-statistic, p = p-value.

*p < .05, †*p *< .001.

Binary logistic regression results indicated that the overall model examining the combined effect of medical fears and covariates on fear-based medical avoidance was significant, *X*^2^(11) = 28.02, *p* = .003. Additionally, the model had an improved goodness-of-fit compared to the null model. The model explained 20.4% (Nagelkerke R^2^) of the variance in medical avoidance and classified 72.5% of cases correctly. However, the model was better at classifying medical care engagement (89.3%) compared to avoidance (36.8%). Despite overall model significance, none of the five MFS-SV dimensions were significant predictors(see Table 4).

**Table 4:** Logistic regression results of medical fear dimensions on medical care engagement (*n* = 180).

Predictor Variable Covariate	*Odds Ratio*	*95% CI*	*p*
Mutilation	1.017	.911 - 1.135	.766
Blood	1.111	.962 - 1.283	.153
Examination/Symptoms	1.061	.953 - 1.181	.280
Injections & Blood Draws	1.143	.995 - 1.313	.058
Sharp Objects	.896	.774 - 1.038	.142
Gender	.231	.063 −.851	.028
Black/African American	.628	.247 - 1.597	.328
Asian	.385	.123 - 1.204	.101
Other race	.530	.166 - 1.694	.284
Ethnicity	1.445	.591 - 3.529	.419
Chronic health status	.945	.429 - 2.082	.888

*Note:* Gender was represented as a dummy coded variable with females serving as the reference group. For dummy coded race variables, white served as the reference group. CI = confidence interval.

## Discussion

The current study assessed the role of different aspects of medical fear in perceptions of provider empathy, provider trust, medical mistrust, and intentions/attitudes toward seeking medical care. We also assessed the impact of medical fears on medical engagement. Results from our study suggested that some dimensions of medical fears were significantly associated with provider and medical trust, as well as perceptions of provider empathy. While none of the five MFS-SV dimensions significantly impacted medical care-seeking attitudes or behaviors, our findings suggested that males were more likely to engage in fear-based medical avoidance than females.

In line with our hypotheses, we found that heightened levels of mutilation fears were associated with lower levels of provider trust and empathy, as well as greater medical mistrust. While no previous studies have directly examined these associations, our findings may be explained by the fact that the mutilation subscale is more highly correlated with measures of disgust sensitivity (i.e., the predisposition of a person to respond to certain stimuli with disgust) [31] than other MFS-SV subscales [19]. Individuals experiencing disgust often perceive others as less trustworthy and have a tendency to make more negative moral judgments [32,33]. Additionally, one study found that when individuals were aware that the source of their disgust was not related to the situation they were making decisions about, the association between trust and disgust ceased [32]. The mechanism through which disgust impacts trust may help explain why fear of mutilation significantly impacted certain study outcomes, whereas the injection/blood draw subscale (also highly correlated with disgust sensitivity) [19] did not. Specifically, fear and disgust related to injections/blood draws may be more readily associated with certain procedures or individuals responsible for those procedures (e.g., phlebotomist, nurse) rather than physicians or the healthcare system; on the other hand, fear of mutilation may be experienced as more ambiguous and, therefore, more broadly associated with medical stimuli.

Conversely, heightened fears of sharp objects and medical examination/symptoms were associated with higher levels of provider trust. There is no previous research specifically linking fear of sharp objects to provider trust. However, it may be that fear of sharp objects is primarily associated with thoughts of an accidental injury perpetrated by someone inexperienced or untrustworthy with sharp objects. Providers may be perceived as having expertise in handling sharp objects and this may alleviate anxieties associated with accidental injury. With regard to medical examination/symptoms, it is notable that this subscale is associated more with anxiety sensitivity than disgust sensitivity [19,20]. Anxiety sensitivity is associated with an inclination to conflate the experience of physical sensations (e.g., dizziness) with sinister causes [19,34]. It is possible that even though individuals fear medical examinations, they are motivated to seek out a trusted medical professional in order to alleviate anxiety associated with their physical symptoms. Experience with medical examinations and visiting providers over the lifespan may also alleviate anxiety related to visits and lead to more provider trust.

Regarding demographic features of our sample, we found that those with chronic illnesses experience medical fears more often than those without chronic illness. While previous research has found high prevalence rates of needle fear among various chronic illness populations in the literature [35], novel findings from our study suggest that people with chronic illness may also be more likely to experience medical fears than those without chronic illnesses. It may be assumed that individuals with chronic illness might have more familiarity with medical procedures and thus experience more trust in the medical system and less fear. However, it is possible that, because of more frequent engagement in medical care, they may actually have a greater risk of exposure to painful procedures that cause and sustain fear. Theoretical models of needle fear development over the lifespan suggest that pain (both the experience and anticipation of it) is one of the key variables that determines the onset and persistent nature of needle fear throughout one’s life [36]. In fact, findings from a recent global study of adults with needle fear further supported this theory, with participants reporting that pain was one of the largest contributing factors to their fear of needle procedures [1]. Thus, more frequent exposure to unmitigated pain during medical procedures may not result in desensitization because conditioned stimuli (e.g., needles) are still paired with an undesirable unconditioned stimulus (pain) that evokes a fear/avoidant response.

Lastly, none of the specific dimensions of medical fear were significantly associated with fear-based avoidance or attitudes toward seeking medical care. There are no previous studies that comprehensively examine all dimensions of medical fear in relation to medical care avoidance. However, the needle fear and BII phobia literature suggests that estimates of fear-based avoidance vary widely across studies and also based on procedure type. For example, a recent international study found that 52% of their sample reported blood draw avoidance and 33% endorsed avoidance of at least one vaccine [1]. Our study asked about any form of medical fear-related health care avoidance rather than avoidance of specific procedures. It is notable that 33% of our participants with medical fears reported some degree of fear-based delay/avoidance of medical care. This finding was somewhat lower than expected based on estimates in the literature. It may be that a lack of prompts with specific procedural examples caused under-reporting or that our sample experienced less severe fear, which resulted in relatively lower rates of medical care avoidance. In sum, factors, such as fainting and severe fear [37], may predict avoidance better than specific dimensions of medical fear. However, these elements alone do not fully explain medical avoidance and future research should continue to examine other contributing factors. We also suggest that future work build off our findings by incorporating more advanced statistical methods, such as path analysis or structural equation modeling, that focus on how constructs may be related to each other in their associations with medical care.

### Limitations

Although this study yielded novel findings, it is not without limitations. The cross-sectional methodology prohibits us from drawing any causal inferences. Additionally, 75.8% of the study sample identified as female and most of our sample was recruited from students currently enrolled in introductory psychology courses. As such, this may impact the generalizability of study results. Furthermore, few studies to date have compared differences in the experience of medical fears across adulthood. This study’s exclusion of individuals in middle and late adulthood may impact the generalizability of the results. Thus, more research is needed that compares differences in the experience of medical fears across adulthood. The emphasis on young adults in our study is a strength given that the prevalence of certain medical fears (i.e., injection/blood draw) is much higher relative to adults 27 + years old [3]. Lastly, internal consistency for the measure of provider trust fell below the accepted value of 0.7 and results using this measure should be interpreted with caution [38].

## Conclusions

Our findings suggest that fear of mutilation is associated with less provider trust and empathy, as well as more medical mistrust. It is well-established in the literature that the quality of patient-provider relationships influences patient outcomes and adherence to medical recommendations [7,8,39,40]. As such, it is vital to implement strategies in medical settings to mitigate distress associated with medical fears. In addition to referrals for cognitive-behavioral treatments, it may be prudent for providers to collaborate with patients to develop treatment plans that take medical fears into consideration. Collaborative and patient-centered approaches are effective at fostering greater communication and trust in patient-provider relationships [39], and may also serve to reduce fear-related distress. Treatment adaptations to address medical fears may include use of pain management techniques (e.g., topical lidocaine), smaller needles, non-invasive treatment alternatives (e.g., nasal spray vaccines), pharmaceuticals, and distractions during procedures [1,6,41,42]. Additional research should examine the impact of medical interventions on reducing distress related to medical fears, and on patient engagement in recommended care.
